# Birth asphyxia related mortality in Northwest Ethiopia: A multi-centre cohort study

**DOI:** 10.1371/journal.pone.0281656

**Published:** 2023-02-24

**Authors:** Daniel Bekele Ketema, Fantu Mamo Aragaw, Fasil Wagnew, Misganaw Mekonnen, Abeba Mengist, Alehegn Aderaw Alamneh, Yihalem Abebe Belay, Getiye Dejenu Kibret, Cheru Tesema Leshargie, Molla Yigzaw Birhanu, Yitbarek Tenaw Hibstie, Belisty Temesgen, Animut Alebel

**Affiliations:** 1 Department of Public Health, College of Health Science, Debre Markos University, Debre Markos, Ethiopia; 2 The George Institute for Global Health, University of New South Wales (UNSW), Sydney, Australia; 3 Department of Epidemiology and Biostatistics, Institute of Public Health, College of Medicine and Health Sciences, University of Gondar, Gondar, Ethiopia; 4 Department of Nursing, College of Health Science, Debre Markos University, Debre Markos, Ethiopia; 5 Department of Statistics, College of Computational Science, Debre Markos University, Debre Markos, Ethiopia; 6 Department of Medical Laboratory Science, College of Health Science, Debre Markos University, Debre Markos, Ethiopia; 7 Department of Human Nutrition and Food Sciences, College of Health Sciences, Debre Markos University, Debre Markos, Ethiopia; 8 Department of Health Systems and Policy, Institute of Public Health, College of Medicine and Health Sciences, University of Gondar, Gondar, Ethiopia; 9 Faculty of Health, University of Technology Sydney, Sydney, Australia; 10 Debre Markos Comprehensive Specialized Hospital, Debre Markos, Ethiopia; Akershus University Hospital, NORWAY

## Abstract

**Background:**

Birth asphyxia is the second leading cause of neonatal death in Ethiopia, next to preterm-associated infections. Understanding the causes of death in asphyxiated newborns will help to design appropriate care. This study identifies predictors of neonatal mortality in asphyxiated newborns in selected hospitals in Northwest Ethiopia.

**Methods:**

An institution-based prospective cohort study of 480 newborns with birth asphyxia was conducted at Debre Markos Comprehensive Specialized Hospital, Shegaw Motta District Hospital, and Injibara General Hospital. All newborns with asphyxia admitted to the neonatal critical care unit from the first of November 2018 to the first of November 2019 were included. Data were obtained prospectively from mothers using an interviewer’s administered questionnaire. The Kaplan-Meier survival curve was used to estimate survival time, and Log rank test was used to compare the survival curves. Bivariable and multivariable Cox proportional hazards models were fitted to identify the independent predictors of mortality in asphyxiated newborns. Adjusted hazard Ratios (AHRs) with 95% Cis (Confidence Intervals) were used to measure the strength of association and test statistical significance.

**Results:**

The overall cumulative incidence of mortality among asphyxiated newborns was 42.29% (95% CI: 38%, 46). Asphyxiated neonates with other comorbidities (sepsis, neonatal anemia) (AHR = 2.63, 95% CI:1.69, 4.10), oxygen saturation of 50–69 (AHR = 4.62, 95% CI:2.55, 8.37), oxygen saturation of 70–89 (AHR = 2.82, 95% CI: 1.80, 4.42), severe Apgar score at one minute (AHR = 1.59, 95% CI:1.12, 2.25), neonates with Hypoxic Ischemic Encephalopathy (HIE) (AHR = 6.12, 95% CI:2.23, 16.75) were at higher risk of mortality.

**Conclusions:**

The mortality rate among asphyxiated neonates remains high, and slightly higher than previous studies. Asphyxiated newborns with other comorbidities, severe Apgar score at one minute, who develop HIE, and low oxygen saturation were at higher risk of death. Therefore, designing appropriate interventions and prevention methods should be considered for identified variables.

## Introduction

According to the World Health Organization (WHO), birth asphyxia is defined as a failure to initiate and sustain breathing at birth [1, 2]. It can happen at any point during the pregnancy, including antepartum, intrapartum, and postpartum periods. However, it most commonly (70%) happened during the postpartum period [3].

Neonatal mortality (NM) is the occurrence of neonatal deaths within the first four weeks of life and is expressed as neonatal deaths per 1000 live births. It can be classified as early (death of neonates < seven days) and late (neonatal deaths between seven and 28 days). Early NM refers to newborns who die within the first week of life, whereas late NM refers to newborns death between seven and 28 days after birth [4, 5].

In 2016, 46% of all under-five mortality were among babies in their first 28 days of life. More than three-fourths (75%) of NM occur in the early neonatal period (age < seven days) [6]. The vast majority of NM occur in developing countries, where access to health care is limited [6]. According to the 2019 Mini Ethiopian Demographic and Health Survey, NM in Ethiopia was reported as 33 deaths per 1,000 live births [7].

Evidence suggests that more than three-fourths (75%) of NM in developing countries are easily preventable with simple and low cost interventions, such as antibiotics for pneumonia and sepsis, sterile blades to cut the umbilical cords using knit caps and kangaroo care to keep babies warm [8, 9]. The United Nations adopted the Sustainable Development Goals (SDGs) in 2015, with the goal of reducing NM to below 12 per 1,000 live births by 2030 [10].To achieve this ambitious plan, the Ethiopian government has also implemented various strategies including the expansion of emergency obstetric and newborn care services aiming to improve the neonatal and maternal health [11, 12]. Despite remarkable progresses in reducing child mortality, NM remains a significant public health concern.

Although birth asphyxia-related NM is a significant public health concern worldwide, developing nations, including Ethiopia, are the most affected. As per previous studies, the death rate among asphyxiated newborns ranged from 13.3% to 40.3% [13–19]. Different maternal and neonatal sociodemographic features, such as obstetric and health-care-related variables (home delivery, lack of ante natal follow up, spontaneous virginal delivery, premature neonates, shock during admission, severe asphyxia) were associated with mortality of asphyxiated neonates [14, 15, 17–20].

Recognizing the unacceptable maternal and child deaths, as well as the long-term neurological consequences of prolonged labor, the World Health Organization issued recommendations in a document on how to prevent prolonged labor during deliveries. These include training of birth attendants in resuscitation skills, risk factors identification, assessing pelvic outlet, diagnosing presentation and position of the baby, assessing descent of the foetal head, recognizing obstructed labor, and vacuum extraction when indicated [2, 21]. Thus, basic resuscitation by birth attendants competent in resuscitation would substantially help reduce deaths from perinatal asphyxia

Identifying predictors of mortality among asphyxiated newborns is vital to develop effective interventions and taking essential measures in a timely manner. However, factors that influence mortality in asphyxiated newborns in Ethiopia are not well investigated. This prospective cohort study was designed to identify predictors of mortality among newborns who suffered from birth asphyxia in selected hospitals in Northwest Ethiopia. The findings of this study will have implications for improving neonatal survival.

## Methods

### Study design, period, and setting

An institution-based prospective cohort study was conducted from the first of November 2018, to the first of November 2019 in selected hospitals in Northwest Ethiopia. The study was conducted at Debre Markos Comprehensive Specialized Hospital, Shegaw Motta District Hospital, and Injibara General Hospital. They are 300 kilometers, 371 kilometers, and 445 kilometers apart from Addis Ababa, Ethiopia’s capital, respectively. The three hospitals serve for more than 6.5 million people in the Amhara regional state and neighboring regions. Apart from other services, all three hospitals offer neonatal intensive care for seriously ill newborns, including those who suffered from asphyxia.

### Study population, sample size and sampling procedures

All newborns with asphyxia admitted to the Neonatal Intensive Care Units (NICUs) of the selected hospitals were the source population. Whereas all newborns with asphyxia admitted to the NICUs between the first of November 2018, and the first of November 2019, were the study population. Neonates with congenital abnormalities (congenital heart disease, hydrops fetalis, structural abnormalities), as well as neonates weighing less than 1 kg at birth were exclude from the study. These neonates were excluded for the following reason: this population is at higher risk of mortality and including them could result in an overestimated NM mortality and leads to biased estimation. Furthermore, neonates admitted without mothers or mothers with psychiatric illnesses were excluded from the study.

The sample size of 441 neonates was calculated using STATA statistical software version 16.0. Based on the assumptions of a 2.04 hazard-ratio associated with hyaline membrane disease [22], with assumed variability of 0.5, a likelihood of death of 0.165 [22], 5% error probability, 0.15 proportion of withdrawal, and 80% power. However, we included all of the newborns (480) who were admitted to the hospitals with hypoxia during the study period. In terms of follow-up, each neonate was followed for a maximum of 28 days. Asphyxiated babies who were discharged before 28 days were contacted by phone to assess the outcome.

### Variables of the study

Neonatal mortality among asphyxiated neonates was the outcome variable for this study. Whereas sociodemographic variables (age of the neonate at admission, sex of the neonate, marital status of the mother, residence, educational status of the mother and occupation of the mother),obstetric and health service related variables (complications at birth, parity, gravidity, antenatal care, place of birth, medical disease during pregnancy), and neonatal related variables (gestational age, other co-morbidities, birth weight, birth type, presence of seizure, HIE, 1-min Apgar score, 5-min Apgar score, oxygen saturation,) were independent variable for this study.

### Operational definitions

**Event:** death of asphyxiated neonates at specific time (day) within the 28 days of follow-up as evidenced by physician confirmation.

**Censored**: asphyxiated neonates who are still alive at the end of follow up, withdrawal and lost-to-follow-up were considered as censored.

**Comorbidities other than asphyxia** includes sepsis, neonatal anemia, hypoglycemia, hypothermia, and so on

Severe Apgar score: a one-minute Apgar score of 0 to 3 or the necessity at birth of positive pressure ventilation.

### Data collection and quality control

Data was collected prospectively using a pre-tested and structured interviewer administered questionnaire. The questionnaire was developed from related literatures and WHO standard verbal autopsy questionnaires [23]. The questionnaire was initially prepared in English and then translated to Amharic language and back to English. Neonates were followed for a total of 28 days. The follow-up process was done in two ways: the first way, data collectors visit the neonate daily, while he/she is in the hospital. The second way, if the neonate discharged from the hospital before 28 days, the data collectors communicate the mother every 7-days using a phone call. When death occurred within 28 days of birth, the date and cause of death had been recorded.

To assure data quality, nurses from each hospital who had been trained on the basic care of NICU and working in the NICU of each hospital were recruited as data collectors. The principal investigators and supervisors closely supervised the data collection procedure. Training about the data collection tool and data collection process was given for data collectors and supervisors for one day at each hospital.

### Data processing and analysis

Data were entered using Epi-Data Version 4.2 and analyzed using STATA Version 16 statistical software. Descriptive statistics including mean, median, and standard deviation were used to describe the neonate cohort characteristics. The Kaplan Meier survival curve, and log rank tests were used to estimate the time to neonatal mortality and compare the categorical variables respectively. Bivariable Cox-proportional hazards regression model were fitted and then, variables with p-value ≤ 0.25 in bivariable analysis were fitted into multivariable analysis. Hazard ratio with 95% confidence interval and p-values were used to measure the strength of association and to identify statistically significant predictors

**Fig 1:**
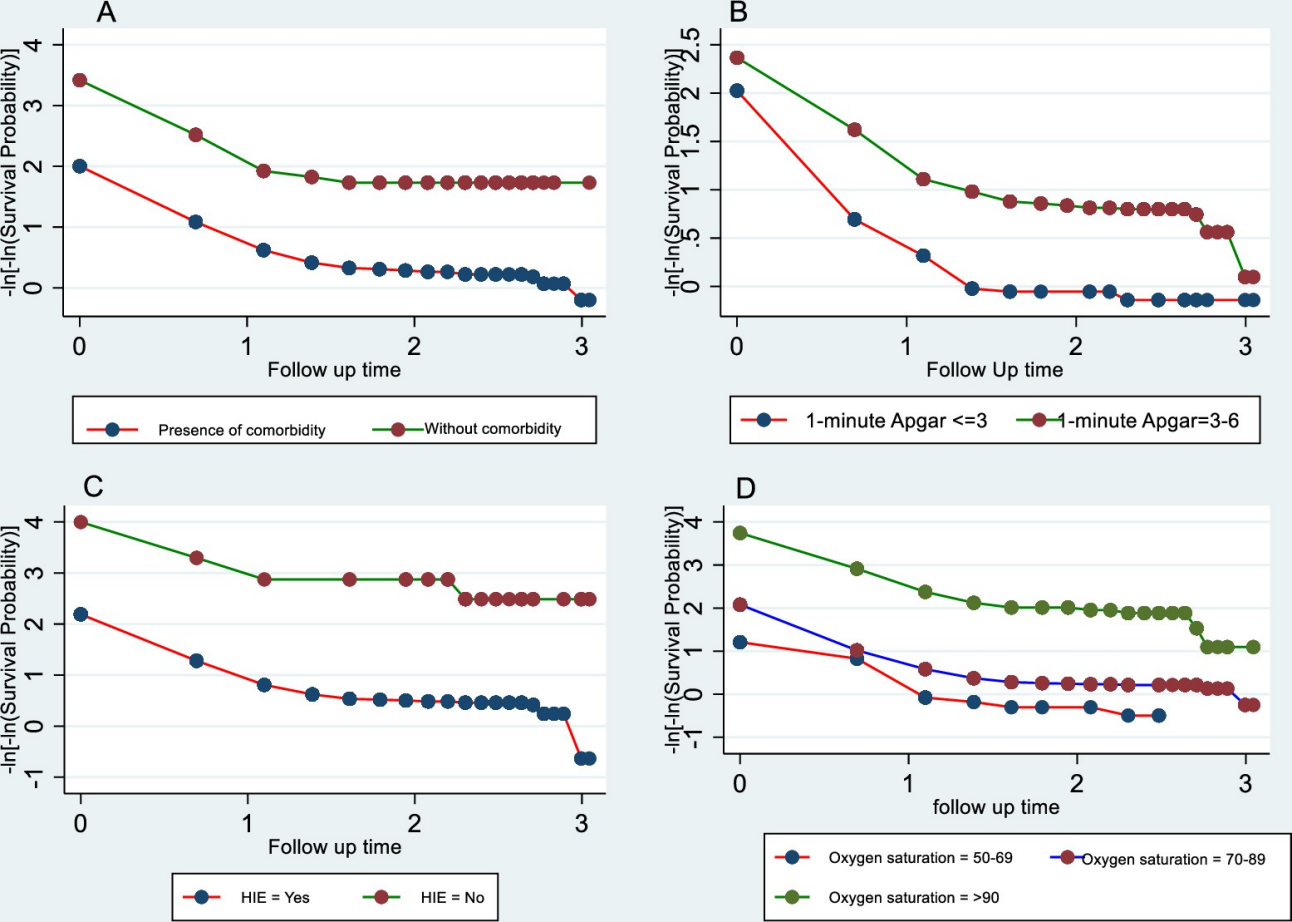
Plot of log (-log (survival probability)) Vs log (analysis time) by comorbidity (A), 1-minute Apgar score (B), Hypoxic Ischemic Encephalopathy (HIE) (C), and level of Oxygen saturation (D).

The proportional hazard assumption was checked using log-log plot, and the plots are parallel which indicates there no violation of the proportionality assumption (**Fig 1**). In addition, a global test based on Schoenfeld residuals found that all the covariates and the full model satisfied the proportional hazard assumption (Chi square  =  25.85, p-value  =  0.26).

**Fig 2:**
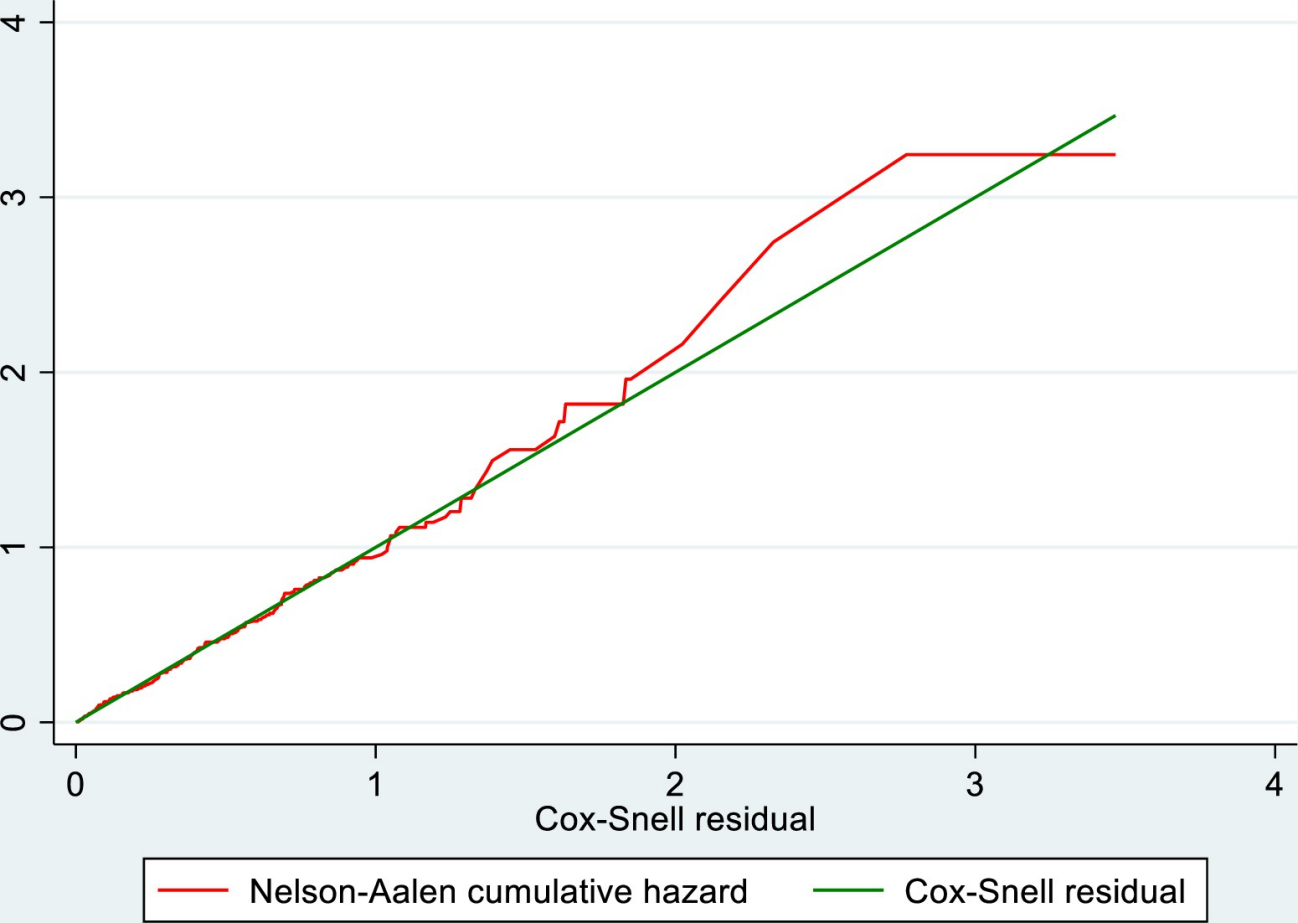
Plot of Nelsen-Allen cumulative hazard function against Cox-Snell residual.

The Cox-Snell residuals were used to assess the model’s quality. As illustrated in **Fig 2**, the hazard function follows the 45-degree line very closely except for increased values of time. It is very common for models with censored data to have some wiggling at increased values of time and it is not something, which should cause much concern. Overall, we would conclude that the final model fits the data very well.

### Ethical considerations

An institutional review committee of Debre Markos University College of Health Science granted ethical approval. A permission letter from each hospital administration was also obtained. Every mother gave verbal informed consent prior to data collection. Furthermore, all collected data were coded and secured in a separate room before being entered into computer to maintain confidentiality. All data were password-protected once entered into the computer. Furthermore, the data-collection format did not include the names of the participants, and the data were not shared with anybody other than the lead investigators.

## Results

### Characteristics of newborn enrolled in the study

During the study period, there were 480 admissions of newborns with asphyxia of which 271 (56.5%) were male. Almost three-quarter (72.5%) of the mothers of asphyxiated neonates were married (**Table 1**). The average gestational age was 38.4 weeks (SD = 2.2), and the mother’s age at the time of the last delivery was 22.0 years (SD = 3.4).

**Table 1:** Newborns and maternal socio-demographic characteristics in selected hospitals of Northwest, Ethiopia.

Variables	Categories	Frequency	Proportion (%)
Sex of the newborn	Male	271	56.5
	Female	209	43.5
Religion of mother	Orthodox Christian	413	86.04
	Muslim	62	12.92
	Other	5	1.04
Marital status of mother	Married	348	72.5
	Divorced	23	4.8
	Single	104	21.7
	Widowed	5	1.0
Occupation of the mother	Housewife	126	26.2
	Farmer	112	23.4
	Government Employee	116	24.2
	Private work	126	26.2
Maternal education level	Not able to read and write	174	36.3
	Primary	123	25.6
	Secondary	116	24.1
	Tertiary	67	14.0
Residence	Urban	342	71.25
	Rural	138	28.75

### Maternal and neonatal clinical characteristics

The majority (81.3%) babies were born at full-term. In terms of mode of delivery, 186 (38.8%) were spontaneous vaginal deliveries, 169 (35.2%) were caesarean sections, and 125 (26.0%) were instrumental deliveries. Four hundred twenty-five (88.5%) babies with birth asphyxia were suffered from Hypoxic Ischemic Encephalopathy (HIE). In terms of HIE stages, 127 (29.9%) had stage I, 210 (49.4%) had stage II, and 88 (20.7%) had stage III hypoxia ischemic encephalopathy. Almost all of the mothers of asphyxiated neonates, 469 (97.7%) had prenatal care follow-up. In terms of delivery location, 41 (8.54%) deliveries were home delivery (**Table 2**).

**Table 2:** New-born and maternal clinical characteristics in Northwest Ethiopia selected hospitals from November 1, 2018, to November 1, 2019.

Variables	Category	Frequency	Proportion (%)
Birth type	Single	466	97.01
	Multiple	24	2.90
Antenatal care	Yes	469	97.70
	No	11	2.30
Pregnancy related complication	Yes	108	22.50
	No	372	77.50
Ever family planning	Yes	394	82.10
	No	86	17.90
Parity	Yes	253	52.71
	No	227	47.29
Contraceptive type (394)	Oral	79	20.0
	Implant	67	17.0
	Injection	248	63.0
Duration of pregnancy	Term	390	81.25
	Preterm	90	18.75
Mode of delivery	Spontaneous delivery	186	38.8
	cesarean section	169	35.2
	Instrumental	125	26.0
Place of delivery	Home	41	8.54
	Health institution	439	91.46
RH status	Positive	407	84.8
	Negative	73	15.2
Mothers TT vaccine	Yes	383	79.8
	No	97	20.2
History of still birth	Yes	48	10.0
	No	432	90.0
Apgar score at one minute	Severe (0–3)	89	18.5
	Moderate (4–6)	299	62.3
	Not documented	92	19.2
Apgar score at five minutes	Sever (0–3)	23	4.8
	Moderate (4–6)	263	75.4
	Not documented	95	19.8
Newborn birth weight (g)	<2500	163	34.0
	> = 2500	317	66.0
Oxygen saturation	50–69	31	6.65
	70–89	264	56.65
	>90	171	36.70
Resuscitation	Yes	342	71.25
	No	138	28.75
Comorbidity other than asphyxia	Yes	325	67.71
	No	155	32.29
Seizure of the neonate during admission	Present	248	51.67
	Absent	232	48.33
HIE	Yes	425	88.50
	No	55	11.50
Stages of HIE (425)	Stage I	127	29.90
	Stage II	210	49.40
	Stage III	88	20.70

RH: Rhesus factor, HIE: Hypoxic Ischemic Encephalopathy, TT: Tetanus Toxoid

### Survival analysis and outcome of the follow up

A total of 480 neonates with asphyxia were observed for varying lengths of time, from one day to 21 days, with a Median follow up duration of seven (IQR = 9) days. This follow-up time contributed a total of 3514-person days of risk under observation. During the study period, 203 (42.3%) of neonate’s deaths were observed, with an incidence rate of 57.7 per 1000 (95% CI: 50.3, 66.3) person-days. Out of 203 total neonatal deaths, 193 (95.07%) died within seven days of delivery. Approximately, 147 (72.4%) of the 203 neonatal deaths had a history of seizure during admission. Mortality among preterm and term asphyxiated neonates was 53.33% and 39.48% respectively.

**Fig 3:**
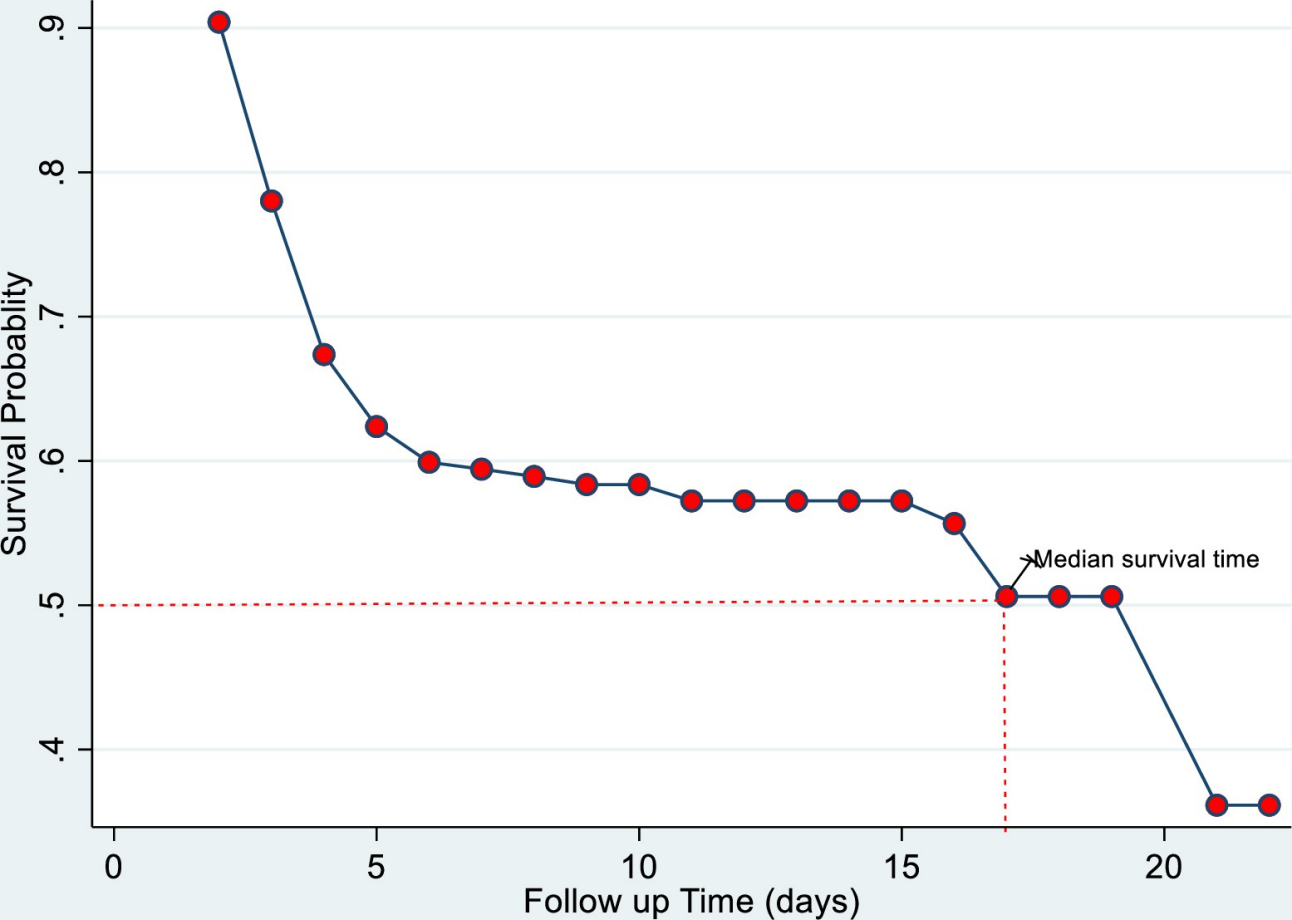
Overall Kaplan-Meier curve of newborns with asphyxia in Northwest Ethiopia selected hospitals from November 1, 2018, to November 1, 2019.

The overall estimated median survival period of asphyxiated neonates was 16 days (95% CI: 7, 20 days) (**Fig 3**), and the cumulative survival probability of a newborn at the end of the study was 49.68 percent (95 percent CI: 42.96, 56.03).

**Fig 4:**
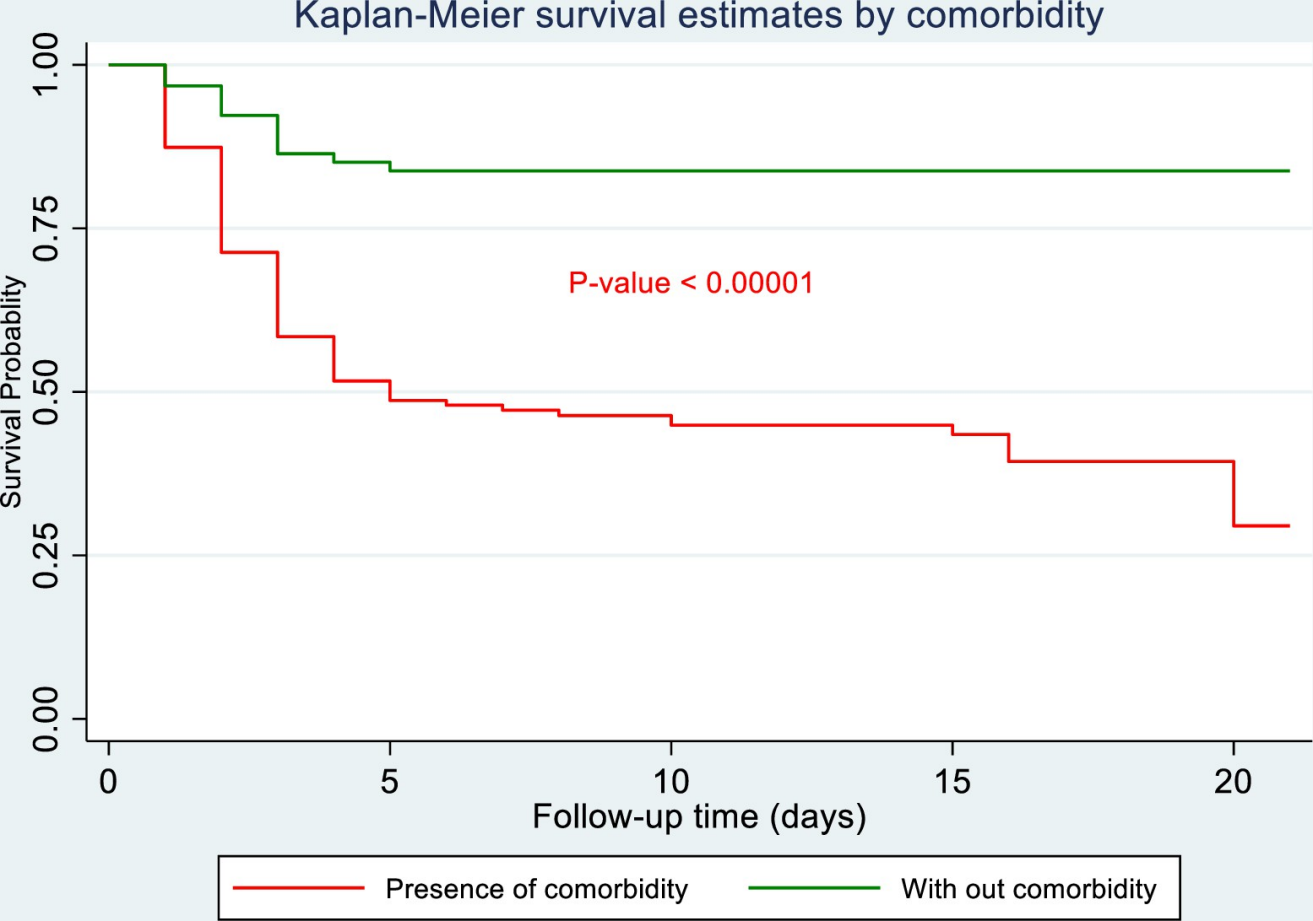
Kaplan-Meier survival curve of newborns with asphyxia by comorbidity in Northwest Ethiopia selected hospitals from November 1, 2018, to November 1, 2019.

The Kaplan-Meier curve demonstrated that newborns with any comorbidity other than asphyxia had a higher risk of mortality and had a shorter survival time (**Fig 4**).

**Fig 5:**
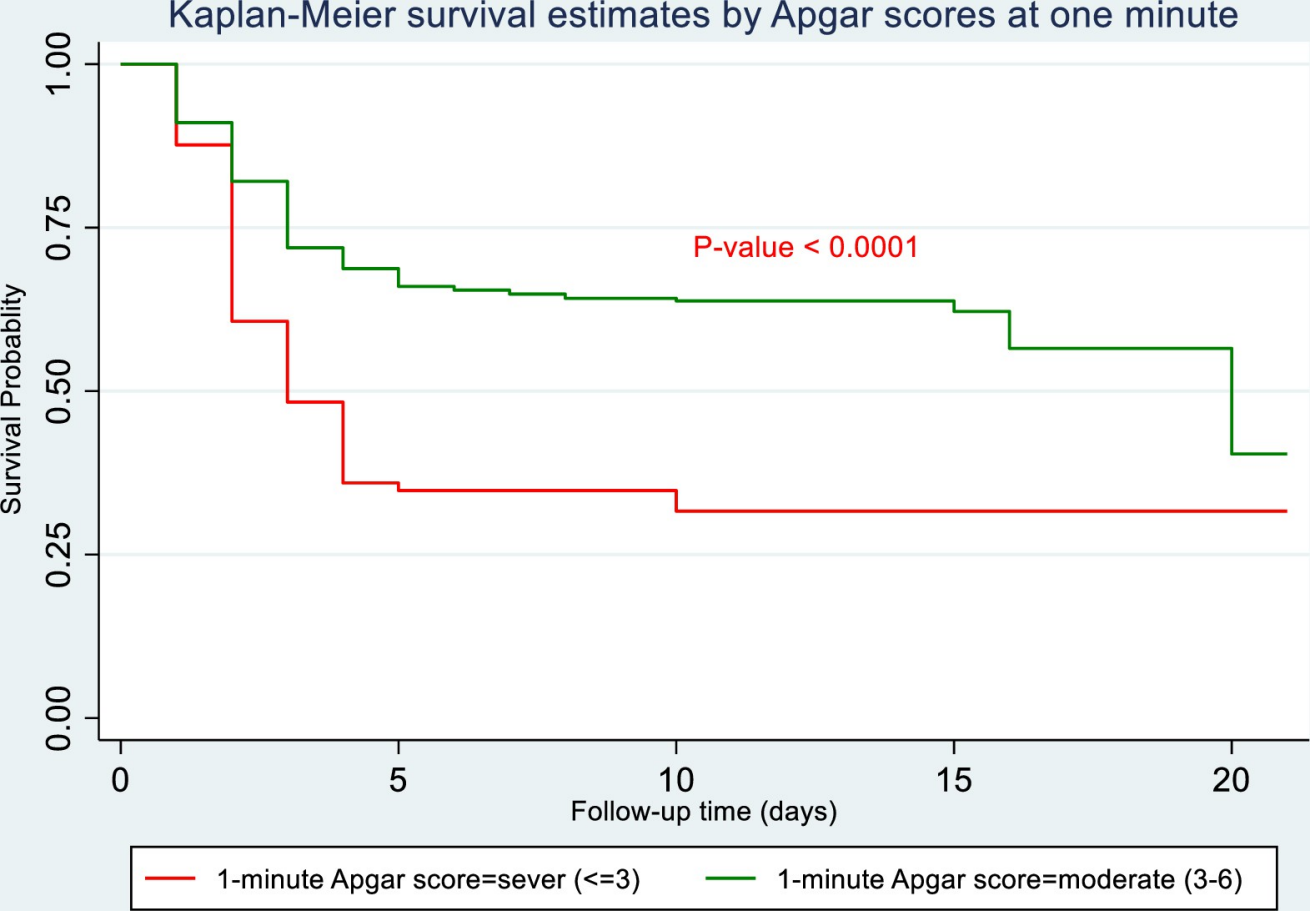
Kaplan-Meier survival curve of newborns with asphyxia in Northwest Ethiopia selected hospitals from November 1, 2018, to November 1, 2019, by 1-minute Apgar score.

Lower Apgar scores at one minute were significantly associated with a higher risk of newborn mortality. As shown in **Fig 5**, neonates with a 1-minute Apgar score of 0–3 were at a higher risk of death than asphyxiated newborns with a 1-minute Apgar score greater than three.

### Predictors of neonatal mortality among asphyxiated neonates

In the bi-variable cox regression analysis, age of mother, maternal education level, occupation of mother, resuscitation, residence, HIE status, birth type, comorbidity other than asphyxia, pregnancy related complication, history of still birth, Apgar score at one minute, Apgar score at five-minute, parity, place of delivery, and oxygen saturation were significant predictors of mortality among asphyxiated neonates with P-values less 0.25.

In the multivariable Cox regression, neonates with comorbidity other than asphyxia had 2.65 times higher risk of dying instantly compared to neonates without comorbidity (AOR = 2.63, 95% CI:1.69–4.10). Neonates with oxygen saturation levels 50–69 had 4.62 times higher risk of death compared to neonates with normal oxgen saturation level (AOR = 4.62,95% CI:2.55, 8.37); whereas neonates with oxygen saturation levels 70–89 had 2.82 times higher risk of death (AOR = 2.82, 95% CI:1.80, 4.42) compared to those neonates with oxygen saturation levels >90. Asphyxiated babies with severe Apgar score at 1 minute are more likely to die than those with a moderate Apgar score (AOR = 1.59, 95%CI: 1.12, 2.25) (**Table 3**).

**Table 3:** Bivariable and multivariable Cox regression analysis of the risk of neonatal mortality among asphyxiated neonates in Northwest Ethiopia selected hospitals from November 1, 2018, to November 1, 2019.

Variables	Categories	Survival status		CHR [95%CI]	AHR [95%CI]
		Event, n [%]	Censored (n, [%]		
**Age of mother**	<20	107 [52.20]	98 [48.80]	1.73 [1.25,2.40]	1.31[0.91, 1.88]
	20–24	55 [35.54]	109 [66.46]	1.00	1.00
	25–29	35 [37.63]	58 [62.37]	1.19 [0.78, 1.82]	1.28 [0.80,2.04]
	>30	6 [33.33]	12 [66.67]	0.96 [0.41, 2.23]	1.02 [0.41,2.49]
**Maternal Education level**	Not able to read and write	85 [48.85]	89 [51.15]	1.56 [0.97, 2.49]	0.85 [0.41,1.75]
	Primary	60 [48.78]	63 [51.22]	1.65 [1.01,2.70]	1.22 [0.62,2.38]
	Secondary	36 [31.03]	80 [68.97]	0.93 [0.55,1.59]	0.97 [0.54,1.75]
	Tertiary	22 [32.84]	45 [67.16]	1.00	1.00
**Occupation of mother**	Housewife	68 [53.97]	58 [46.03]	2.28 [1.50, 3.48]	1.27 [0.64,2.52]
	Farmer	48 [42.86]	64 [57.14]	1.74 [1.11, 2.73]	1.10 [0.53,2.28]
	Government employed	32 [27.59]	84 [72.41]	1,00	1.00
	private work	55 [43.65]	71 [56.35]	1.71[1.11, 2.65]	1.02 [0.58,1.79]
**Residence**	Urban	82 [35.19]	151 [64.81]	1.00	
	Rural	121 [48.99]	126 [51.01]	1.49 [1.13, 1.98]	1.12 [0.70,1.78]
**Resuscitation**	yes	128 [37.43]	214 [62.57]	1.00	1.00
	No	75 [54.35]	63 [45.65]	1.68 [1.26,2.24]	1.36 [0.97,1.90]
**HIE**	Yes	199 [46.82]	226 [53.18]	8.90 [3.28,24.14]	6.12[2.23,16.7]*
	No	4 [7.27]	51 [92.73]	1.00	1.00
**Birth type**	Single	189 [41.45]	267 [58.55]	1.00	1.00
	Multiple	14 [58.33]	10 [41.67]	1.49 [0.87,2.58]	1.17 [0.65,2.12]
**Comorbidity**	Yes	178 [54.77]	147 [45.23]	4.17 [2.74, 6.35]	2.63[1.69,4.10] *
	No	25 [16.13]	130 [83.87]	1.00	1.00
**Pregnancy complication**	Yes	56 [51.85]	52 [48.15]	1.39 [1.02, 1.89]	1.26 [0.90,1.76]
	No	147 [39.52]	225 [60.48]	1.00	1.00
**History of still birth**	Yes	30 [62.50]	18 [37.50]	1.75[1.19, 2.59]	0.85[0.54, 1.34]
	No	173 [40.05]	259 [59.95]	1.00	1.00
**1-minute Apgar score**	Severe	60 [67.42]	29 [32.58]	2.19 [1.62,2.97]	1.59[1.12,2.25] *
	Moderate	143 [36.57]	248 [63.43]	1.00	1.00
**5-minute Apgar score**	Severe	15 [65.22]	8 [34.78]	1.95 [1.15, 3.31]	1.37 [0.75, 2.50]
	Moderate	188 [41.14]	269 [58.86]	1.00	1.00
**Parity**	Prim gravida	93 [36.76]	160 [63.24]	1.00	1.00
	Multigravida	110[48.46]	117 [51.54]	1.40 [1.06, 1.85]	1.23 [0.88,1.72]
**Place of delivery**	Home	23[56.10]	18 [43.90]	1.58 [1.02,2.44]	
	Health institution	180[41.00]	259 [59.00]	1.00	1.00
**Oxygen saturation**	50–69	23 [74.19]	8 [25.81]	8.11 [4.58, 14.34]	4.62[2.55,8.37] *
	70–89	147 [55.68]	117 [44.32]	4.77 [3.12,7.31]	2.82[1.80,4.42] *
	>90	25 [14.62]	146 [85.38]	1.00	1.00

*Statistically significant variables; HIE Hypoxic Ischemic Encephalopathy

**CHR**: Crude Hazard Ratio; **AHR**: Adjusted Hazard Ratio

## Discussion

Neonatal mortality rate remains unacceptably high in resource-limited countries including Ethiopia. This study was designed to determine the birth asphyxia mortality rate and its predictors in selected hospitals in Northwest Ethiopia. In this study, the overall cumulative incidence of mortality among asphyxiated newborns was 42.29% (95% CI: 38%, 46). This finding is higher than previous studies from developing and developed countries elsewhere [19, 24–27], and in consistent with findings from Nigeria and Sri Lanka [28, 29]. The variation in neonatal mortality might be attributed to low quality resuscitation practice and inadequate thermal control. As evidence suggested inadequate thermal control contributed to neonatal death in NICU despite the provision of resuscitation [30].

The greater risk of infant mortality in our study may be justified by the fact that, despite the seeming adequate quantity of care provided for resuscitation of asphyxiated neonates, the quality might have been substandard. This finding implies, need to develop proper care and monitoring systems during and immediately after birth by properly trained and qualified healthcare personnel.

In agreement with previous literature [24, 31–33], this study found that asphyxiated babies with a severe Apgar score at 1 minute were at higher risk of death than those with a moderate Apgar score. This could be explained by the fact that a low Apgar score indicates fetal distress, which appears to be a worsening clinical state and eventually death. As a result, neonates with an Agar score of 0–3 in the first minute of life require pulmonary support and the most appropriate resuscitation strategy. In addition, this finding will be primarily explained by the fact that tissue oxygenation, which is a function of respiratory rate, heart rate, and oxygen saturation, is a key determinant of hypoxia and, as a result, hypoxic encephalopathy in perinatal asphyxia [20].

We found that asphyxiated babies with low oxygen saturation had a higher risk of death. This finding was in support of existing literature [20, 34]. These increased chances of death when oxygen saturation levels fall could be due to insufficient oxygen supply, which can cause severe hypoxia ischemic organ damage resulting in a fatal outcome [35].

This study found that asphyxiated neonates with Hypoxic Ischemic Encephalopathy (HIE) were more likely to die than asphyxiated newborns without HIE. This finding was consistent with earlier researches [13, 25, 29, 36]. This could be because HIE is usually associated with either mild or severe organ damage in asphyxiated babies, resulting in an elevated neonatal mortality rate. Moreover, birth asphyxia combined with other comorbidities was associated with a higher mortality as supported by other studies [37].

Our research has limitations. Some cases throughout the study period were not followed because they were lost to follow up, which could have an impact on the estimated newborn mortality rate. The cause of death for the discharged neonate was not confirmed by the physician, which may have resulted in an overestimation of the parameter. Moreover, other neonatal treatments were not investigated as factors, which may preclude the true effect of other variables.

## Conclusions

According to the findings of this study, the overall mortality rate among asphyxiated infants was high. Newborns with comorbidities other than asphyxia, a severe Apgar score at one minute, neonates who develop HIE, and neonates with low oxygen saturation were identified as having the highest risk of neonatal death. Therefore, designing appropriate care and prevention methods should be considered for these identified variables.

## Supporting information

S1 Data (DTA)

## List of abbreviations

NICU: Neonatal Intensive Care Unit
HIE: Hypoxic Ischemic Encephalopathy
SDGs: Sustainable Development Goals
AHR: Adjusted hazard Ratio
NM: Neonatal mortality
TT: Tetanus Toxoid
WHO: World Health Organization
SD: Standard Deviation
IQR: Interquartile Range

## References

[1] World Health Organization: Guidelines on basic newborn resuscitation available at http://www.who.int/maternal_child_adolescent/documents/basic_newborn_resuscitation/en/. 23700652

[2] Enweronu-Laryea C, Dickson KE, Moxon SG, Simen-Kapeu A, Nyange C, Niermeyer S, et al: Basic newborn care and neonatal resuscitation: a multi-country analysis of health system bottlenecks and potential solutions. *BMC pregnancy and childbirth* 2015, 15(2):S4. doi: 10.1186/1471-2393-15-S2-S4 26391000 PMC4577863

[3] Martinez-Biarge M, Diez-Sebastian J, Wusthoff CJ, Mercuri E, Cowan FM: Antepartum and intrapartum factors preceding neonatal hypoxic-ischemic encephalopathy. *Pediatrics* 2013:peds. 2013–0511. doi: 10.1542/peds.2013-0511 24019409

[4] The partnership for maternal and child health: Newborn death and illness available at http://www.who.int/pmnch/media/press_materials/fs/fs_newborndealth_illness/en/. 2015.

[5] Jehan I, Harris H, Salat S, Zeb A, Mobeen N, Pasha O, et al: Neonatal mortality, risk factors and causes: a prospective population-based cohort study in urban Pakistan. *Bulletin of the world Health Organization* 2009, 87(2):130–138. doi: 10.2471/blt.08.050963 19274365 PMC2636189

[6] World Health Organization: Newborns: reducing mortality available at http://www.who.int/news-room/fact-sheets/detail/newborns-reducing-mortality. 2018.

[7] Central Statistical Agency [Ethiopiaphic): Demographic and Health Survey Key Indicators available at https://dhsprogram.com/pubs/pdf/PR81/PR81.pdf. 2016.

[8] UNITED NATIONS: The Millennium Development Goals reports available at http://www.un.org/millenniumgoals/2015_MDG_Report/pdf/MDG%202015%20rev%20%28July%201%29.pdf. 2015.

[9] Federal Ministry of Health of Ethiopia: Neonatal Intensive Care Unit (NICU) Training Participants’ Manual unpublished report 2014.

[10] UNITED NATIONS: The 2030 agenda for Sustainable Development available at https://sustainabledevelopment.un.org/content/documents/21252030%20Agenda%20for%20Sustainable%20Development%20web.pdf. In.

[11] Kayongo M, Rubardt M, Butera J, Abdullah M, Mboninyibuka D, Madili M: Making EmOC a reality—CARE’s experiences in areas of high maternal mortality in Africa. *International Journal of Gynecology & Obstetrics* 2006, 92(3):308–319. doi: 10.1016/j.ijgo.2005.12.003 16442536

[12] Mekbib T, Kassaye E, Getachew A, Tadesse T, Debebe A: The FIGO save the mothers initiative: the Ethiopia–Sweden collaboration. *International Journal of Gynecology & Obstetrics* 2003, 81(1):93–102. doi: 10.1016/s0020-7292(03)00071-7 12676407

[13] Yelamali B, Panigatti P, Pol R, Talawar K, Naik S, Badakali A: Outcome of newborn with birth asphyxia in tertiary care hospital-a retrospective study. *Medica Innovatica* 2014, 3(2):59–64.

[14] Trotman H, Garbutt A: Predictors of outcome of neonates with hypoxic ischaemic encephalopathy admitted to the neonatal unit of the University Hospital of the West Indies. *Journal of tropical pediatrics* 2010, 57(1):40–44. doi: 10.1093/tropej/fmq040 20525776

[15] Yadav S, Shah GS, Poudel P, Mishra OP: Risk factors for adverse outcome in asphyxiated new born in Eastern Nepal. *International Journal Of Community Medicine And Public Health* 2017, 3(6):1419–1423.

[16] Agrawal J, Yadav S, Chaudhary S, Kafle S: Treatment and outcome of neonates with hypoxic ischaemic encephalopathy at BP Koirala Institute of Health Sciences. *Sri Lanka Journal of Child Health* 2017, 46(2).

[17] Seyal T, Hanif A: Factors related to adverse outcome in asphyxiated babies. *Annals of King Edward Medical University* 2009, 15(4):180–180.

[18] Adebami OJ: Maternal and fetal determinants of mortality in babies with birth asphyxia at Osogbo, Southwestern Nigeria. *Glo Adv Res J Med Med Sci* 2015, 4(6):270–276.

[19] Padayachee N, Ballot DE: Outcomes of neonates with perinatal asphyxia at a tertiary academic hospital in Johannesburg, South Africa. *South African Journal of Child Health* 2013, 7(3):89–94.

[20] Ekwochi U, Asinobi NI, Osuorah CD, Ndu IK, Ifediora C, Amadi OF, et al: Incidence and Predictors of Mortality Among Newborns With Perinatal Asphyxia: A 4-Year Prospective Study of Newborns Delivered in Health Care Facilities in Enugu, South-East Nigeria. *Clinical Medicine Insights*: *Pediatrics* 2017, 11:1179556517746646. 10.1177/1179556517746646 PMC5734560 29276422

[21] Ali A, Masakhwe BA: WHO midwifery education module 3 managing prolonged and obstructed labour. *Training Course in Sexual and Reproductive Health Research* 2010.

[22] Orsido TT, Asseffa NA, Berheto TM: Predictors of Neonatal mortality in Neonatal intensive care unit at referral Hospital in Southern Ethiopia: a retrospective cohort study. *BMC pregnancy and childbirth* 2019, 19(1):1–9. 30819143 10.1186/s12884-019-2227-5 PMC6396455

[23] Organization WH: Standard neonatal verbal autopsy questionnaire. Revised version. World Health Organization Geneva, WHO 2003.

[24] Cavallin F, Menga A, Brasili L, Maziku D, Azzimonti G, Putoto G, et al: Factors associated with mortality among asphyxiated newborns in a low-resource setting. *The Journal of Maternal-Fetal & Neonatal Medicine* 2020, 35(6):1–6. 10.1080/14767058.2020.1743670 32212882

[25] Egharevba O, Kayode-Adedeji B, Alikah SJJon-pm: Perinatal asphyxia in a rural Nigerian hospital: incidence and determinants of early outcome. *Journal of neonatal-perinatal* 2018, 11(2):179–183. 10.3233/NPM-1759 29966208

[26] Biselele T, Naulaers G, Bunga Muntu P, Nkidiaka E, Kapepela M, Mavinga L, et al: A descriptive study of perinatal asphyxia at the University Hospital of Kinshasa (Democratic Republic of Congo). *ournal of tropical pediatrics* 2013, 59(4):274–279. doi: 10.1093/tropej/fmt011 23486392

[27] Joseph S, Bindusha S, Radhika S, Krishnan R, Kumar SJIJoCH: Clinical profile and short-term outcome of perinatally asphyxiated term neonates in a tertiary hospital in Southern Kerala. *Indian Journal of Child Health* 2017, 4(3):399–404.

[28] Uleanya ND, Aniwada EC, Ekwochi UJAhs: Short term outcome and predictors of survival among birth asphyxiated babies at a tertiary academic hospital in Enugu, South East, Nigeria. *African health sciences* 2019, 19(1):1554–1562. doi: 10.4314/ahs.v19i1.29 31148983 PMC6531974

[29] Meshram RM, Bokade CJSLJoCH: Risk factors for mortality in birth asphyxia of outborn neonates: a prospective observational study. *Sri Lanka Journal of Child Health* 2019, 48(1):26–32.

[30] Lunze K, Bloom DE, Jamison DT, Hamer DH: The global burden of neonatal hypothermia: systematic review of a major challenge for newborn survival. *BMC medicine* 2013, 11(1):1–11. 23369256 10.1186/1741-7015-11-24 PMC3606398

[31] Araya T, Ghiwot H, Gidey G, Tilahun W, Mola MJR, Medicine RAJo: Risk Factors of Neonatal Deaths Among Asphyxiated Neonates in Ayder Referral Hospital, Mekelle, Ethiopia: A Case Control Study. *Research & Reviews*: *Journal of Medicine* 2015, 5(3):16–26.

[32] Andegiorgish AK, Andemariam M, Temesghen S, Ogbai L, Ogbe Z, Zeng LJBph: Neonatal mortality and associated factors in the specialized neonatal care unit Asmara, Eritrea. *BMC public health* 2020, 20(1):1–9. 31907008 10.1186/s12889-019-8118-x PMC6945585

[33] NDAYISENGA TJIJoM, Health P: Maternal and newborn risk factors associated with neonatal mortality in Gitwe district hospital in Ruhango district, Rwanda. *International Journal of Medicine and Public Health* 2016, 6(2).

[34] Schmidt B, Whyte RK, Asztalos EV, Moddemann D, Poets C, Rabi Y, et al: Effects of targeting higher vs lower arterial oxygen saturations on death or disability in extremely preterm infants: a randomized clinical trial. *Jama* 2013, 309(20):2111–2120. doi: 10.1001/jama.2013.5555 23644995

[35] Golubnitschaja O, Yeghiazaryan K, Cebioglu M, Morelli M, Herrera-Marschitz M: Birth asphyxia as the major complication in newborns: moving towards improved individual outcomes by prediction, targeted prevention and tailored medical care. *EPMA Journal* 2011, 2(2):197–210. doi: 10.1007/s13167-011-0087-9 23199149 PMC3405378

[36] Amritanshu K, Smriti S, Kumar V, Pathak A, Banerjee DPJJoCN: Clinical profile and short-term outcome of hypoxic ischemic encephalopathy among birth asphyxiated babies in Katihar medical college hospital. *Journal of Clinical Neonatology* 2014, 3(4):195.

[37] Dongol S, Singh J, Shrestha S, Shakya AJJoNPS: Clinical profile of birth asphyxia in Dhulikhel Hospital: A retrospective study. *Journal of Nepal Paediatric Society* 2010, 30(3):141–146.

